# Early development of abstract language knowledge: evidence from perception–production transfer of birth-language memory

**DOI:** 10.1098/rsos.160660

**Published:** 2017-01-18

**Authors:** Jiyoun Choi, Anne Cutler, Mirjam Broersma

**Affiliations:** 1 Hanyang Phonetics and Psycholinguistics Lab, Hanyang University, Seoul, South Korea; 2 Max Planck Institute for Psycholinguistics, Nijmegen, The Netherlands; 3 ARC Centre of Excellence for the Dynamics of Language, Australia; 4 The MARCS Institute, Western Sydney University, New South Wales, Australia; 5 Centre for Language Studies, Radboud University, Nijmegen, The Netherlands

**Keywords:** phonological acquisition, speech perception–production link, abstract representation, international adoptees, retention of early knowledge

## Abstract

Children adopted early in life into another linguistic community typically forget their birth language but retain, unaware, relevant linguistic knowledge that may facilitate (re)learning of birth-language patterns. Understanding the nature of this knowledge can shed light on how language is acquired. Here, international adoptees from Korea with Dutch as their current language, and matched Dutch-native controls, provided speech production data on a Korean consonantal distinction unlike any Dutch distinctions, at the outset and end of an intensive perceptual training. The productions, elicited in a repetition task, were identified and rated by Korean listeners. Adoptees' production scores improved significantly more across the training period than control participants' scores, and, for adoptees only, relative production success correlated significantly with the rate of learning in perception (which had, as predicted, also surpassed that of the controls). Of the adoptee group, half had been adopted at 17 months or older (when talking would have begun), while half had been prelinguistic (under six months). The former group, with production experience, showed no advantage over the group without. Thus the adoptees' retained knowledge of Korean transferred from perception to production and appears to be abstract in nature rather than dependent on the amount of experience.

## 1. Introduction

The process of acquiring a language starts both automatically and extremely early—before birth, during the last third of the time a baby spends in the womb. By that time, assuming successful development of hearing and of memory construction abilities, the baby is able to segregate one stream of sound reaching the womb from sounds with other sources [1], recognize a repeatedly experienced auditory input [2] and as a result also recognize the mother's voice [3]. This early experience lays a foundation for a lifetime of speaking and listening to the native language. At birth, we indeed prefer to listen to the language heard in the environment rather than to another language [4], and from later infancy on throughout life, no matter how many languages we eventually experience, the native language may enjoy an advantage in many aspects of language use, including resistance to noise-masking [5], and recognition of already heard talkers [6–8].

The first year of life is thus a period of astonishing linguistic development. Both the ability to distinguish the phonemic contrasts of a native language and the ability to pick out and store words appear during this period, and decades of research on these processes have produced an emerging consensus that they develop in parallel [9]. Accurate isolation of words is supported by the knowledge that certain sounds are contrastive in the native language, and learning of the language's inventory of contrastive sounds is supported by creation of sound–concept pairings. In their early months, infants can distinguish more sound contrasts than the native language actually uses, and it is only towards the end of the first year of life that they show a more adult-like tendency to discriminate the native language contrasts more efficiently than contrasts of another (non-native) language [10], suggesting that compilation of the native phonemic inventory is being mastered. In the same period, a rapid increase occurs in the number of words they can recognize and, by the end of the first year, they begin to produce words too. Word recognition is not a simple consequence of the contrasts being compiled, though, because even by around six months infants show recognition of spoken words referring to familiar people or objects in their environment [11,12]. And although speaking intelligible words appears and gladdens the parental heart around age 11–12 months, in fact during the preceding half year infants' prelinguistic vocalizations gradually take on ever more features of the native language, both its phonemes [13] and its rhythm and intonation [14].

The nature of the knowledge about sounds and words that accrues during this time, and in particular, the knowledge which supports the transition from listening to speech to also producing speech, is an issue of lively debate in language development research. Do infants build up a stock of memory traces of speech perception experience [15], with their early productions being aimed at replicating these traces, or do they compute abstract generalizations from what they hear [16], with their productions being aimed at these generalized targets? In adulthood, the perception–production link shows clear evidence of being mediated by abstract phonological representations. For instance, in speech repetition, production is determined by phonological factors such as phoneme identity, but not by details of pronunciation such as the talker's accent [17]. Does very early speech production then also rely on abstractions, or must the development of abstraction be treated as a later stage in language acquisition?

In this study we address this question. We draw on a recently appreciated source of evidence concerning what is retained from early linguistic experience, which has indicated that at least perceptual representations formed in infancy can persist for years without benefit of further input. This evidence concerns children who were born in one country but adopted at an early age by a family in another country, with a quite different language. In their case, the language input that was heard in the womb and in the early months of life does not correspond to the language that eventually becomes the native language, the language in which education is attained and the language in which the user, as a child and as an adult, communicates most comfortably. These international adoptees commonly acquire their new language swiftly and effectively [18,19] and within a year or two have mastered it at least as well, sometimes better than, their local peers [20,21]. Their birth language, by contrast, is rapidly forgotten. Adoptees who as young children could speak in their birth language start within months to forget words [22–24] and by adulthood report no memories at all of the language they first began to learn [25,26]. Thus, the linguistic work that these children carried out in those vital early months of life may perhaps seem to have gone to waste.

Nonetheless, there are many tantalizing indications that memory of early language learning is preserved, such that it can usefully be accessed if ever needed. Some such evidence has come from studies of an analogous situation, namely heritage-language exposure (where older family members use a language that the youngest generation hears but does not speak). When students with exposure to such a heritage language enrol in a course to formally learn that language, they often prove to outperform their classmates both in perception of the language-specific speech contrasts [27,28] and in production of the same sounds [27,29]. Although in the heritage-language case these benefits can be in part ascribed to continuing exposure to the language, benefits of early input have also been observed without continuing exposure; for instance, English learners of Hindi who had been exposed to the language in infancy (but not since then) displayed better discrimination of Hindi consonant contrasts than their classmates with no early exposure [30].

Disruption of exposure is of course more drastic in the case of international adoptees. A study using the language-student model as in the heritage-language studies [27–29] assessed the performance of learners of Korean after about 2 years of study [31] but found no difference in phonological perception between students who were adoptees from Korea and students without any such early exposure. If there is no difference after 2 years of study, it may be that phonological benefits will reveal themselves most notably in the facility with which initial (re)learning occurs. Indeed, there are some strong indications of such a particular initial learning benefit. A study in which adult native English-speakers received short-term training on non-English phonemic contrasts from Hindi or Zulu revealed that adults who had been exposed to Hindi or Zulu in childhood showed greater improvement on this task than control participants without the childhood experience [32]. Similarly, children adopted from India into English-speaking families in America performed indistinguishably from control children on perception of a phoneme contrast before explicit training, but significantly better than the control group after a short discrimination training session [33].

In our own laboratory, too, we have discovered a relearning benefit with adoptees from Korea who received phonetic identification training for Korean consonant contrasts; before training, these adoptees and a control group performed equally poorly, but the effect of training was to increase identification performance quite rapidly for the adoptees but significantly more slowly for the control group [34,35]. With heritage learners, there is evidence that their exposure to two different phonetic systems (rather than to a single system) may more effectively attune the perceptual system to speech sound processing in general [36]. Such a skill-based explanation for our perception results could be rejected, however, because the same two groups performed equivalently on simple same–different judgement tasks with familiar and unfamiliar sounds from different languages [37]. Though more finely honed skills in general may arise given sustained concurrent exposure, sequential exposure to separate systems, as has been the experience of international adoptees, does not have this effect. The relearning benefit shown by adoptees thus indeed seems to be based on knowledge acquired in early life, prior to their switch to a new language, even though that knowledge is no longer consciously accessible.

Neuropsychological evidence confirms that early learning indeed leaves traces of such knowledge. Although prior work had shown similar activation patterns in adult adoptees and monolingual adults who listened to sentences in the language of the environment versus the adoptees' birth language [25], a more recent study [38] with a discrimination task produced a different result; 10- to 17-year-olds adopted as infants from China, whose adopted (and only functional) language was Canadian French, showed brain activation patterns like those of Chinese--French bilinguals when discriminating Chinese lexical tones, while monolingual French-speakers who had never been exposed to Chinese had quite different activations. Even though the adoptees had no working knowledge of their birth language at all, their brain retained the ability to interpret its tonal contrasts linguistically.

The existence of linguistic knowledge that is laid down very early in life is not surprising, given the evidence of language-specific knowledge at birth and during the earliest months thereafter. No matter how early a baby is adopted, there will have been some exposure to the first language and some learning that supports the formation of linguistic representations. The existence of such representations at the time of adoption is unremarkable; what is suggested by the evidence reviewed above, however, is that such representations are by no means lost, but are retained and remain useful if an occasion for their use arises.

Our successful test [34,35,37] of the relearning benefit proposal for perception accompanied the present study, involving the same participants. We here address the nature of unconsciously retained linguistic representations, by going beyond the perceptual evidence for a relearning advantage and focusing now on the perception–production transfer. We investigate, in other words, whether these demonstrated benefits in perception are accompanied by speech production benefits. As noted, heritage-language learners, who have enjoyed the advantage of continued exposure to perceptual models, have proved better in producing as well as perceiving sounds [27,29], but as yet there is no similar evidence from adoptees without exposure to their birth language post adoption. Such evidence can provide important insights into exactly what is retained from the early experience, and in consequence into the formation in infancy of linguistic representations. If abstract phonological representations form the basis of the linguistic knowledge retained by adoptees, then transfer of a perceptual relearning benefit to a production benefit should be possible.

Our participants were adult citizens of The Netherlands with Dutch as primary language of use and with no conscious knowledge of Korean; the adoptee group were all adopted from Korea early in life. Among the latter we compared a subset (approximately half the group) who were adopted early in the first year of life, when they would not yet have been able to produce intelligible speech, and another subset (the other half) who were aged 17 months or older when adopted, and hence would already have been deploying a medium-sized early vocabulary. Although at the time of testing no participant could produce any words of the birth language, a history of early production in the language versus no such production experience is potentially an important factor.

In comparison with prior studies our participant group was large (58: 29 adoptees and 29 matched controls). We did not adopt the approach of those studies testing students already enrolled in a language course; given the evidence that the retained benefits are best observed in an initial relearning phase, we used short-term perception training across a period of less than 2 weeks. We offered no training in production of the unfamiliar sounds, but instead recorded our participants' ability to imitate the perceptual training targets early in training, and at its completion.

The perceptual training [34,35] involved a Korean contrast with no analogue in Dutch or English. Where the latter two languages have a feature contrast that distinguishes voiced versus voiceless stop consonants, Korean has no voiced stops but a three-way distinction in the articulatory dynamics of voiceless stops. As is clear from decades of research [39] on the difficulty of the English /r/-/l/ distinction for listeners with only one phoneme in the /r/-/l/ space, a novel distinction that divides perceptual space more finely than expected is very hard for both speakers and listeners. The measure of production success was identification scores and goodness rating by native Korean listeners in Seoul.

## 2. Methods

### 2.1. Participants

All participants (Korean adoptees, Dutch control participants, Korean control participants) had completed at least high school, and none reported hearing, speaking or reading disability. All were paid for participating.

The adoptee group comprised 29 Korean-born Dutch speakers (age 23–41 years, *M* = 32 years, 21 females) who were adopted by Dutch-speaking families between the ages of 3 and 70 months (5 years and 10 months, henceforth 5;10, *M* = 1;10). The population of adoptees from Korea in The Netherlands consists of an earlier wave, who were mostly adopted as toddlers, and a later wave, who were mostly adopted under 6 months of age (the latter group were also mostly female). We incorporated this group difference into our design. Fifteen participants were adopted after 17 months of age (eight females, range = 1;5–5;10, *M* = 3;3, mean years since adoption 32, mean age at test 34;7) and 14 below 6 months (13 females, range = 0;3–0;5, *M* = 0;4, mean years since adoption 28, mean age at test 28;4). None had learned Korean after adoption. Approximately half of the adoptees (*N* = 16) had made short visits to Korea since adoption (from 9 to 28 days); the majority visited once (*N* = 12), though a few had made two (*N* = 3) or three (*N* = 1) trips. All adoptees were recruited by contacting the Dutch Association for Korean Adoptees Arierang and by word of mouth.

The Dutch controls were 29 native speakers of Dutch (age 19–47 years, *M* = 32 years, 16 females) who had not learned Korean. The controls were as closely matched as possible to the adoptees in terms of six control variables that might influence learning performance in the study: (i) age at the time of testing, (ii) highest completed level of education among the four levels in the Dutch high school system (from lowest to highest: VBO, MAVO, HAVO, VWO), (iii) history of visiting Korea (for adoptees, post adoption), (iv) mean ratio of how long the participant stayed in Korea to how long ago they visited Korea, (v) number of languages participants knew (if only a little), and (vi) sex. There were no significant differences between the adoptees and controls on any variable (see electronic supplementary material, table S1 for these analyses). Half of the controls (*N* = 15) were either siblings (*N* = 9) or partners (*N* = 6) of Korean adoptees and were recruited in a similar way as the adoptees; the other half had no such relationship. Among the latter group, eight had made a short visit to Korea and were recruited through word of mouth (giving 15 controls in total with such a visit). The remaining six controls were from the Max Planck Institute for Psycholinguistics participant pool.

At the outset, adoptees and controls were asked about their reasons for participating. The groups replied similarly, the majority reporting ‘To help research’ (adoptees 29%, controls 32%) or ‘Out of interest’ (adoptees 34%, controls 18%), with some controls (18%) stating ‘For partners or siblings’.

The adoptees' and controls' conscious knowledge of Korean was assessed with a Korean-childhood-vocabulary recognition test following the main experiments (see electronic supplementary material, text S1 for the procedure). No significant difference in proportion correct was found between the groups (adoptees: *M* = 0.46, s.d. = 0.17; controls: *M* = 0.48, s.d. = 0.11; *t*_56_ = 0.55, *p* = 0.59).

A control group of 25 native speakers of Korean (age 27–37 years, *M* = 30 years, 14 females) was recruited by flyers posted at Hanyang University, Seoul. These participants provided native control data for both the perception and production studies. Sixty more native Koreans (age 19–36 years, *M* = 24 years, 16 females) were recruited in the same way to assess the productions, as were 10 Korean native speakers (age 22–33 years, five females) who recorded the perceptual training stimuli.

### 2.2. Materials

Where English has a two-way contrast between voiceless and voiced stop consonants (*two*, *do*; *core*, *gore*; and *pay*, *bay* at the alveolar, velar and bilabial places of articulation, respectively), and Dutch has the same, Korean has three realizations of such consonants. They are known as fortis, lenis and aspirated stops, and just as with the English two-way contrast, sets of minimally differentiated common words abound. The three versions of the alveolar stop, for instance, occur in the minimal set 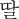 [t*ar] ‘daughter’, 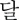 [tar] ‘moon’ and 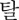 [t^h^ar] ‘mask’. All three are voiceless in the pre-vocalic position; they differ from one another in voice onset time (VOT), and in the fundamental frequency (F0) in the transition to a following vowel. Fortis stops are distinguished from lenis and aspirated stops by a distinctively short VOT, and the latter two are distinguished by degree of aspiration and by F0, with low F0 for the lenis versus high F0 for the aspirated. Dutch, by contrast, distinguishes voiceless versus voiced stops, whereby the primary cue for the distinction is VOT; voiced stops can have negative VOT, or ‘pre-voicing’ (vocal fold vibration before the release burst), or close to zero VOT, while voiceless stops have positive VOT. Note that Dutch voiceless stops (in contrast with English voiceless stops) are not aspirated, and for Dutch listeners, the presence versus absence of VOT is the distinctive cue [40]. For second-language learners, the hardest type of target-language contrast to master is indeed one that divides a phonetic space more finely than the learner's language does [39,41]. Given that Dutch has only one voiceless stop category, whereas Korean has three, the Korean contrast should be difficult for Dutch speakers.

In the perceptual part of the project (see [34,35,37]), these materials were used to train the adoptees and Dutch control groups on the three-way contrast in the alveolar stops ([t*, t, t^h^]). Training stimuli were 25 minimal triplets of Korean disyllabic CVCV (C: consonant; V: vowel) pseudo-words, differing only in the word-initial consonant. The initial stops were followed by the initial vowels [a], [e], [i], [o] or [u] and then the second syllables [ra], [he], [mi], [t∫o] or [su]. These 75 items (25 triplets) were initially recorded by each of 10 native speakers of standard South Korean, who read the items in a clear citation style. This training (over a period of 10–12 days) consisted of 13 sessions of six practice trials and 75 training trials each, using a three-alternative forced-choice identification task with feedback. Early sessions (1–10) contained stimuli from one (different) talker; later sessions (11–13) had multiple talkers. Order of the sessions was fixed across participants, while the stimuli in each session were presented in random order. Participants completed the training at a quiet location (home or workplace) chosen by themselves. The experimenter (J.C.) visited the participants four times during that period, on average every 2.3 days; between visits, participants undertook training sessions alone, using equipment provided (a laptop and high-quality headphones). All sessions were fully logged on the computers and all participants completed the programme as instructed. Perceptual test sessions were carried out at the outset of training, midway during the training, and finally.

Further details of the perceptual training, and the full results, can be found in [34,35]. As described above, the results showed evidence of retained knowledge in that the adoptee group improved their perceptual identification performance to a greater extent than the controls. In keeping with the proposal [32,33] that relearning ability is where such a benefit is primarily observed, the most marked adoptee–control difference was found between outset and the midway point. The two adoptee subsets (adopted before 6 months of age, adopted after more than 1 year of age) both exhibited the relearning benefit with neither showing it to a greater degree than the other, and age at adoption did not correlate with any perceptual measure. Thus the relearning benefit appeared insensitive to the absolute amount of prior linguistic exposure.

### 2.3. Procedure

#### 2.3.1 Production recording

The adoptees and Dutch controls were recorded twice at the same location as the training: during the first training session (Time 1) and in the final session, i.e. after the entire perceptual training had been completed (Time 2). The native Korean-speaking controls (given no perceptual training) were recorded once at Hanyang University, Seoul.

The stimuli included examples of the alveolar stops used in the perceptual training as well as velar and bilabial stops varying over the same three-way contrast (the latter sets provide a measure of generalization of learning). Nine triplets of Korean CV pseudo-words that differed only in lenis, fortis and aspirated stops were created: three triplets for each of the alveolar, bilabial and velar places of articulation. The vowel was [a], [i] or [u]. All 27 items (nine triplets) were recorded by a female native speaker of Seoul Korean (34 years old) who did not contribute training stimuli.

A rapid-repetition task was used, in which participants heard stimuli one at a time and were required to repeat each stimulus immediately. Each trial started with a fixation mark in the centre of the computer screen for 200 ms, followed by an auditory stimulus. Participants had 2 s to respond. The stimuli were blocked by item, giving 27 blocks of three trials each. The order of the blocks was fixed across participants, starting with the alveolar stop series in [t*a], [t*i], [t*u], [ta], [ti], [tu], [t^h^a], [t^h^i], [t^h^u] order, followed by bilabial and finally velar stops in corresponding order. This 27-block sequence was iterated three times. Participants were informed whether they should produce a lenis, fortis or aspirated stop by presentation of a corresponding symbol on the computer screen. Recordings were made with a Zoom Handy Recorder H2 and a Rode SVM N 3594 microphone at a sampling rate of 44.1 kHz. Each recording lasted approximately 15 min.

#### 2.3.2. Production assessment

We selected one token of each item from each recording time from each speaker, yielding 3807 tokens in all (29 adoptees × 27 items × 2 times + 29 Dutch controls × 27 items × 2 times + 25 Korean controls × 27 items × 1 time). In principle, we chose the last token of the second repetition; if the token was disqualified due to background noise or clipping distortion, the last token (128 tokens; 3.4% of all tokens) or the first token (10 tokens; 0.3% of all tokens) of the third repetition was used instead. The selected tokens were arranged in three sets, each containing 1269 tokens with the same vowel ([a], [i] or [u]). Each set thus had all nine stops (three-way contrast, three places of articulation) from all speakers at each time.

Two independent groups of 30 native listeners of Korean assessed the productions. One group (age 19–36 years, *M* = 24 years, eight females) identified the tokens; the other (age 19–30 years, *M* = 24 years, eight females) rated the goodness of the stop's realization. All were tested individually with a laptop running Presentation software (v. 14.7, Neurobehavioral Systems Inc.) and high-quality headphones, in a testing room at Hanyang University, Seoul. In each group, 10 participants were randomly assigned to each of the three sets (with [a], [i], [u]).

##### 2.3.2.1.Identification task

Participants were asked to listen carefully to each CV stimulus and to identify the consonant as lenis, fortis or aspirated by pressing one of three keys on the computer keyboard (three-alternative forced choice). Each trial began with an empty screen for 600 ms, followed by an auditory stimulus and a screen with the three consonant options in Korean orthography. Participants had 10 s to respond. In each set, stimuli were blocked by place of articulation, in the fixed order alveolar, bilabial, velar; within blocks, order of stimuli was randomized. Each block began with an instruction screen stating the three consonant response options for the coming block, in Korean orthography, followed by six practice trials without feedback. A short break occurred between blocks; the experiment in total took about 35 min.

##### 2.3.2.2.Rating task

Participants were asked to listen carefully to the consonant sound of each CV stimulus and to rate the consonant's pronunciation (by pressing number keys on the computer keyboard) on a scale of 1 (very poor) to 4 (very good). Each trial started with an empty screen for 600 ms, followed by a display of a target consonant and a 4-point scale with descriptions in Korean at each scale point. The auditory stimulus was then presented and participants had 10 s to respond. The stimuli were blocked by the target consonant, in the fixed order [t], [t*], [t^h^], [p], [p*], [p^h^], [k], [k*], [k^h^]; within blocks, stimuli were randomized. Prior to each block, participants were informed of the target consonant for that block and given 10 practice trials. A short break was taken after every three blocks; the experiment again took about 35 min.

## 3. Results

### 3.1. Production assessment results

We calculated mean (arcsine transformed) proportions of correct identification responses and mean ratings for by-listener and by-speaker analyses. Trials with response times over 10 s were excluded, as were trials for which no response option was chosen (together 183 trials; 0.23% of data). Repeated-measures ANOVAs were computed on each dependent variable, with Group (Adoptee, Dutch control), Time (1, 2), Place of articulation (Alveolar, Bilabial, Velar), Phonation type (Aspirated, Fortis, Lenis) and Vowel (/a/, /i/, /u/) as independent variables. We first discuss the results that directly test our research questions (i.e. main effects of Group and of Time, and an interaction between Group and Time).

#### 3.1.1. Identification accuracy: adoptees versus Dutch controls

**Figure 1.**
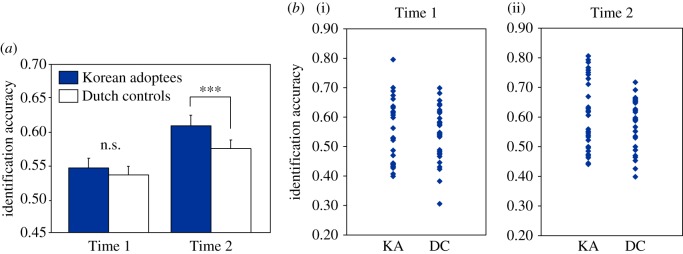
(*a*) Mean proportion of correct identification responses for Korean adoptees' versus Dutch controls' productions at Times 1 and 2 (error bars represent standard errors; ***p < 0.001) and (*b*) proportion of correct identification responses at individual speakers' first (i) and second (ii) production time points as a function of group (KA, Korean adoptees; DC, Dutch controls).

Figure 1*a* displays identification accuracy by group. Accuracy was comparable across groups at the first production times, but higher for adoptees than for Dutch controls at the second production times. A by-listener ANOVA showed significant main effects of Group (*F*_1,27_ = 29.9, *p* < 0.001) and Time (*F*_1,27_ = 156.9, *p* < 0.001), and an interaction between Group and Time (*F*_1,27_ = 5.6, *p =* 0.025). Follow-up analyses revealed this interaction to be due to significantly more accurate categorizations of adoptees' than of Dutch controls' Time 2 productions (*F*_1,27_ = 23.8, *p* < 0.001), but no significant difference in categorization accuracy between the groups' Time 1 productions (electronic supplementary material, figure S1*a* shows individual identification accuracy by Time). The accuracy for both groups' Time 1 productions was, however, above chance level (adoptees: *t*_28_ = 7.6, *p* < 0.001; Dutch controls: *t*_28_=5.4, *p* < 0.001). By-speaker analyses yielded a significant main effect of Time (*F*_1,56_ = 19.8, *p* < 0.001) only, with no significant main effect of Group (*F*_1,56_ = 1.0, n.s.) or Group–Time interaction (*F*_1,56_ = 1.0, n.s.). It is apparent from figure 1*b* that there are large variations in accuracy across speakers within each group, giving rather large standard deviations that might explain this lack of significance.

The percentages correct were, as expected, highest for the trained (alveolar contrasts). The results for the velar and bilabial contrasts were analysed separately to ascertain whether generalization of the three-way distinction to other places of articulation was differently achieved across groups. A by-listeners ANOVA across Group, Time and Place of articulation with two levels (Bilabial, Velar) produced significant main effects of Group (greater identification accuracy for adoptees' productions: *F*_1,29_ = 18.8, *p* < 0.001) and of Time (higher accuracy for Time 2 productions: *F*_1,29_ = 53.1, *p* < 0.001). There was no main effect of Place of articulation and no Group by Time interaction. A significant three-way interaction among all variables (*F*_1,29_ = 4.4, *p* < 0.05), however, revealed that, for bilabial targets, adoptees' productions received significantly higher correct responses than Dutch controls' productions at both Time 1 and 2, whereas for velar targets, the adoptee advantage appeared only for Time 2 productions.

Analysis of the nature of identification errors revealed a similar pattern across all sets of productions, with approximately half of all erroneous identifications being accounted for in all cases by two, of the possible six, error types (lenis heard as aspirated; fortis heard as lenis); this suggests that the strongest factor in determining the listeners' choice when they made an error was acoustic similarity. For this reason the pattern of error types for adoptee and Dutch control productions (at each time point) and with native Korean productions was positively correlated; nonetheless, the ordering of error types for the adoptees' productions by Time 2 very strongly resembled the ordering of error types for native Korean productions. For more details on this analysis, see electronic supplementary material, text S2.

#### 3.1.2. Ratings: adoptees versus Dutch controls

**Figure 2.**
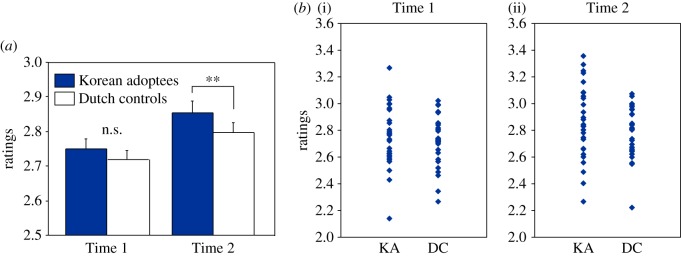
(*a*) Mean ratings for adoptees' versus Dutch controls' productions at Times 1 and 2 (error bars represent standard errors; **p < 0.01) and (*b*) ratings at individual speakers' first (i) and second (ii) production times as a function of group (KA, Korean adoptees; DC, Dutch controls).

As can be seen in figure 2*a*, the pattern for ratings was similar to that for identification: ratings were higher for the adoptees' than for controls' second production times, while there was little difference at the groups' first times. The by-listener analysis showed significant main effects of Group (*F*_1,27_ = 9.6, *p* = 0.005) and Time (*F*_1,27_ = 119.2, *p* < 0.001) with no interaction between the two. Again, however, adoptees' utterances at Time 2 were significantly more highly rated than those of the Dutch controls (*F*_1,27_ = 13.4, *p* = 0.001), whereas there was no significant group difference for Time 1 (electronic supplementary material, figure S1*b* shows individual ratings by Time). The by-speaker analysis produced a significant main effect of Time (*F*_1,56_ = 15.2, *p* < 0.001) but no main effect of Group (*F*_1,56_ = 0.7, n.s.) or Group–Time interaction (*F*_1,56_ = 0.3, n.s.). As with identification accuracy, the non-significance here could be attributed to the wide variance among individuals in each group (figure 2*b*).

The ratings for the bilabial and velar productions were again examined separately to ascertain whether generalization across place of articulation had occurred (the trained alveolar productions were, as expected, again rated most highly). A by-listeners ANOVA as for the identification data revealed significant main effects of all three factors: Group (higher ratings for adoptees: *F*_1,29_ = 12.8, *p* < 0.01), Time (higher ratings for Time 2 productions: *F*_1,29_ = 17.6, *p* < 0.001) and place of articulation (higher ratings for velar targets: *F*_1,29_ = 18.1, *p* < 0.001), but no interactions.

#### 3.1.3. Comparison with Korean control speakers

Identification accuracy and ratings for the Time 2 productions of the adoptees and Dutch controls were compared (in *t*-tests) with those for the utterances contributed by Korean control speakers, separately for each target consonant. All comparisons were significant, indicating that the Korean controls' productions (with a mean identification accuracy across targets of 0.89 and mean ratings of 3.54) were significantly more accurately identified and more positively rated than adoptees' or Dutch controls' productions for all consonants (*p*'s < 0.001; see electronic supplementary material, text S3 for further control analyses).

#### 3.1.4. Effect of age at adoption

Correlations were computed between adoptees' age at adoption and the identification accuracy and ratings at each of their production times. An initial comparison examined whether adoption age correlated with any control factor or with scores in the Korean-childhood-vocabulary test. This revealed a significant negative correlation between adoption age and age at test (*r* = −0.59, *p* = 0.001), indicating that the higher the adoption age, the higher is the current age. Adoption age was also significantly associated with sex (*t*_27_ = 3.38, *p* = 0.002), such that the adoption age was significantly higher for the male (*M* = 3;4) than the female participants (*M* = 1;3). To control for these related factors, we computed partial correlations between the adoption age and each performance measure for the adoptees' productions, separately for each time point. No significant correlations were found.

In ANOVAs (across listeners and speakers) with the variable Adoption age (early, later), the early-adopted group actually performed significantly better than the later-adopted group (accuracy by listener: *F*_1,27_ = 148.5, *p* < 0.001; accuracy by speaker: *F*_1,27_ = 5.8, *p* = 0.023; ratings by listener: *F*_1,27_ = 107.0, *p* < 0.001; ratings by speaker: *F*_1,27_ = 4.6, *p* = 0.041), with no interactions between Adoption age and Time. However, as just noted, the subgroups differed significantly in age at test (*t*_27_ = 4.0, *p* < 0.001) and sex ($\chi_{1}^{2}=5.7$, *p* = 0.017), the early-adopted subgroup having a lower current age and a higher proportion of females (13/14 versus 8/15); see electronic supplementary material, table S2 for all between-subgroup comparison statistics. In an analysis of covariance (ANCOVA) across speakers with current age and sex as covariates, the effect of Adoption age disappeared (and did not interact with other factors). Consistent with the correlational results, we conclude that there is no performance effect of age at adoption.

### 3.2. Relation between perception and production

Finally, we conducted correlation analyses between the perceptual results and the present production results. A complete correlation matrix is provided in electronic supplementary material, table S3 (see also text S4 for further correlations incorporating control factors from hierarchical regression analyses). For the Dutch control participants, there were no systematic effects of the perception–production relationship, although weak correlations appeared between Time 2 production scores and final perceptual scores in particular. For the adoptees, however, there were strong and systematic positive correlations for both production measures at each of the two sampling points with all three perceptual test results, as well as with the degree of improvement across the whole perceptual training, and even with the degree of improvement in the first half of the training, where the adoptee relearning benefit had been most visible.

**Figure 3.**
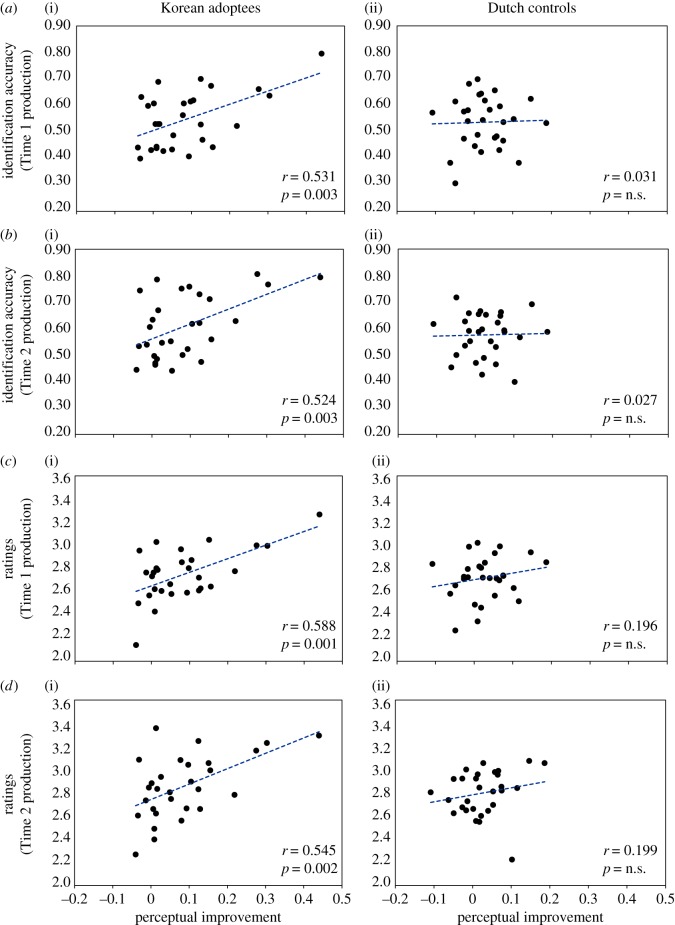
Scatter plots with regression lines comparing improvement across participants in perceptual identification accuracy from pre- to midway test (*x*-axis) against (*a*) Time 1 identification accuracy, (*b*) Time 2 identification accuracy, (*c*) Time 1 ratings and (*d*) Time 2 ratings (*y*-axis) for individual speakers, separately for adoptees (i) and Dutch controls (ii). Pearson correlation coefficients and corresponding *p*-values are shown in the bottom right of each plot. For the adoptees only, perceptual improvement correlates significantly with all production assessment results.

This latter correlation of the relearning benefit in the perception results (difference between the pretest and the midway test; *x*-axis) with the various production assessment results (*y*-axis) is plotted, separately for the adoptees and Dutch controls, in figure 3.

The inter-group difference can be clearly seen in the figure: the adoptees show positive correlations between perceptual improvement and all production assessment results, while the controls show no such positive correlations. Note that removing one adoptee who showed the largest perceptual improvement reduced only the correlation with the Time 1 identification accuracy (to *r* = 0.36, *p* = 0.062).

We conducted correlations comparing production improvement from Time 1 to Time 2 with the perceptual improvement scores, across individuals, and these may be found in electronic supplementary material, table S3. For the Dutch controls, there were again no significant effects at all (all *p*'s > 0.1). For the adoptees, all correlations were positive, but only two reached even near-significance: total perceptual improvement during training with the rated goodness improvement (*r* = 0.35, *p* = 0.063) and perceptual improvement in the latter part of training with rated goodness improvement (*r* = 0.48, *p* < 0.005). Again, we point to inter-participant variability across the group in interpreting the weakness of this individual-level effect.

## 4. General discussion

The benefits that have been widely reported for adoptees' perception of the sounds of their birth language also transfer to production. Native listeners identified the target sounds produced by the adoptee group more accurately than the sounds produced by the controls, and they rated the goodness of the adoptees' productions more highly also.

Both groups of participants in our study significantly improved their perceptual identification scores over the full course of the 13 training sessions [34,35], and both groups likewise improved their production scores from the first to the second production time reported here, as evidenced in both the identification and rating results. But just as the adoptees had significantly outperformed the control participants in the speed with which they learned to perceive the presented sounds [34,35], so did they outperform the controls in the degree to which their productions of these sounds became more identifiable and more highly rated. This advantage held for both the late-adopted subgroup (who would have had at least some experience of speech production in the birth language) and the early-adopted subgroup (who would have not even been babbling speech sounds at the time of their adoption).

These findings have important implications for our understanding of the general questions addressed by our study, namely: what is the nature of the knowledge that is formed in the earliest stages of language development (which is, here, the knowledge that has been retained by the adoptees), and what is the relationship between early speech perception and early speech production? With respect to the first question, we call attention to the fact that the adoptees significantly outperformed the Dutch controls in successfully generalizing the three-way contrast they had learned to places of articulation not used in the training sessions; their bilabial and velar pronunciation attempts were identified more successfully and were more highly rated. As pointed out by Bowers *et al.* [32, p. 1068], such generalization suggests that the knowledge involved is not low-level detail, but has been abstracted from the particular listening experience. Abstractly represented knowledge can then be applied to newly encountered instances.

Further, a crucial aspect of the present study (and also its perceptual counterpart) must be reckoned to be the subgroup results, i.e. the equivalent performance of adoptees who were exposed to their first language only for less than 6 months, versus those who were adopted later and had a year and a half or more of exposure to Korean. The first group would never have produced Korean words, while the latter group would once have had a nascent productive vocabulary, and would have settled upon the native phoneme contrast inventory. The (few) very oldest members among the later-adopted group may have known hundreds of words and have been talking in sentences at the time of adoption. Yet, remarkably, none of that additional experience translated in the present study to an advantage over their earlier-adopted peers.

As described in the introduction, the current consensus on the early development of language-specific knowledge about sounds and words is that there is no hard-and-fast ordering (mastery of an initial phonemic repertoire necessarily preceding construction of an initial vocabulary, or vice versa); instead, there is evidence that the two skills are developed in tandem [9]. In the past few years of infant speech perception research, the earliest appearance of word-level recognition behaviours has been steadily pushed back before the age of 6 months [11,12,42], although, as described in the introduction, at 6 months infants do not yet exhibit language-specificity in their phoneme discrimination [10]. The implication of this is that the necessary underlying learning is well underway in the first half year of life, and would have been available to infants adopted in that period of their life.

The speech input that infants are presented with will support development of a phonological system in that systematic evidence will accrue in support of the speech sounds that belong to the system in question. Other sounds may also be heard—mispronunciations or allophonic variants or effects of external influence on speech production—but in an unsystematic manner. Concentrating upon the contrasts supported by the most systematic evidence will allow the young learner to settle upon the correct repertoire. Among this systematic evidence available in a Korean language environment will be occurrences of the same vowel following three stop consonants that share a constant place of articulation but vary across three possible settings of their articulation dynamics. Our investigations show that knowledge derived from early speech input containing this three-way articulatory contrast has been retained by our adoptee participants, despite cessation of relevant evidence from speech input.

The nature of infants' early linguistic knowledge cannot be directly ascertained by any available means, and the major modelling approaches in infant language development interpret it in different terms. Exemplar models ([15] for development; cf. [43] for adult speech perception) hold that linguistic knowledge consists of veridical traces of speech episodes; in such a model, what is retained in the present instance might be seen to be such traces of originally heard words containing the contrasts. Distributional learning models [44–46] propose that more abstract distributional patterns are computed from speech input; here the statistical patterns of the birth language might be held to be retained by our adoptees, alongside the patterns computed for the later-encountered language. Abstractionist models of various flavours ([47,48]; cf. [49,50] for adult speech perception) propose that knowledge of the abstract structures of a language is computed from the input and retained. This knowledge can be of many kinds, such as syntactic relationships, articulatory patterns or phonetic contrasts; in such an approach, the adoptees' retained knowledge might consist, for example, in the potential for the phonetic contrast repertoire to contain a three-way articulation dynamics contrast.

Given the complete absence of a difference in the presence or accessibility of the retained knowledge between the earlier- and later-adopted groups, i.e. the absence of any effect of the absolute amount of early exposure, any model based on pure exposure accrual, as exemplar-based models are, seems not to be supported by our data. Rather, in line with modelling approaches that allow a role for different kinds of knowledge in development, the more appropriate conclusion should invoke the retention of knowledge not necessarily *of* the words and sounds in question, but rather *about* the nature of the contrast. This could take the form of a generalization across a distribution (as in a distributional learning model) or of the concept that a three-way distinction is allowable (as in an abstractionist model).

In the light of this conclusion, we turn next to the implications for the perception–production relationship. The production of intelligible speech does not happen without perceptual exposure, and in that sense at least these mental operations are closely related; a recent review [51] shows that this relationship is clearly underpinned in neural architecture. The acquisition of difficult perceptual second-language distinctions between consonants is known to produce parallel improvements in perception and in production (see [52] for data from Japanese children learning the English /l/-/r/ distinction across a 1-year period). A tightly coupled interrelationship with perception in fact forms part of production models of many different flavours, including episodic theory [43], direct action [50], abstractionist [53], and learning-based [54], and a recent special issue devoted to this relationship [55] has provided much new evidence of interdependence.

One of these studies [56] concerned, as the present study does, perception and production of a three-way phonetic contrast not present in the participants' language (adult Spanish-speakers were trained on a three-way fricative/affricate contrast of Basque). Successful perceptual learning was interfered with when participants also produced the target sounds during training. The multiple task aspect (perception and production in a single learning trial) was held to be key to the deleterious effect, and suggests a very tight inter-operational dependence. Interestingly, existing (albeit imperfect) knowledge of the language contrast significantly attenuated this interference effect: a group with self-reported intermediate knowledge of Basque (listening, speaking, reading and writing) showed less disruption from additionally required production than did groups without this knowledge. The amount of knowledge involved in [56] was of course immensely greater than that of the present adoptee participants, who self-reported no knowledge of Korean at all, and could not reliably identify even the simplest early-vocabulary items from their birth language. Nevertheless, common to both studies is that underlying existing knowledge of language contrasts is able to support a more constructive perception–production relationship.

Evidence for a role for abstract knowledge in speech production has long been available [57] in data from speech errors, and continues to accrue (e.g. speech errors show effects of phonotactic pseudo-constraints newly learned from perception [58]). The transfer of heard pronunciations to production in an imitation task [17] has been shown to depend on abstraction from the perceptual experience. Some models of initial speech learning are based on the necessary, and inevitably abstract, coupling of articulation to audition [48], and infant cognition shows many and varied types of evidence for abstract knowledge, both non-linguistic [59,60] and linguistic [16,47,61]. Our demonstration that the perceptual relearning effects of adoptees' retained knowledge parallel an advantage in speech production accuracy, and can, moreover, be generalized to newly encountered instances, is therefore fully compatible with our conclusion that the nature of the retained linguistic information is not veridical memory traces of accrued exposures, but rather an abstraction from those early exposures.

In conclusion, our study has provided the first evidence that residual knowledge of the birth language by international adoptees not only helps them relearn a perceptual discrimination, but also supports more efficient speech production. Effects of stored knowledge in production as well as in perception had previously been observed in heritage-language learners acquiring fuller mastery of a family language which they had heard a lot but never previously spoken [27,29]; however, such learners presumably receive better and perhaps more varied perceptual models and have had recent reinforcement of these, which could make their production targets more accurate than those of talkers without such continued exposure. The present study has subjected the participants' productions to the rigorous test of native-user judgements. The listeners' ratings and identifications could not have been influenced by any knowledge of the independent variables under investigation, as the stimuli they received were grouped only by phoneme identity and not by any other factor: productions from each test time and from all three groups (adoptees, Dutch controls, Korean controls) were randomly mixed in the input they heard. The results were highly orderly in each task, with the native Korean control productions receiving appropriately high ratings and identification accuracy, and the Dutch speakers, whether adoptee or control, scoring significantly less well. Crucially, however, the differences within that latter group, between the adoptees and their matched controls, were consistent and striking. Effects of retained knowledge are thus visible both in perceptual relearning and in production, not only for the knowledge received from perception by heritage learners, but also for the knowledge abstracted and retained by international adoptees from their very early but then wholly discontinued linguistic exposure. Equally informative was the finding that even our youngest adoptee group showed evidence of this knowledge retention, suggesting that important and lasting cognitive abilities are being laid down even in the earliest months of life.

## Supplementary Material

Supplementary Information

## Supplementary Material

Data file: Identification accuracy and ratings for Korean adoptees', Dutch controls', and Korean controls' productions.

## Data accessibility

The datasets supporting this article have been uploaded as part of the electronic supplementary material.
