# Supplementary material for: Early development of abstract language knowledge: evidence from perception–production transfer of birth-language memory
Source: R Soc Open Sci. 2017 Jan 18;4(1):160660. doi: 10.1098/rsos.160660 (PMC5319333; doi:10.1098/rsos.160660)
Supplement: Supplementary Information [file rsos160660supp1.docx]

**Supplementary Information**

**Table S1**. Statistics for comparison between Korean adoptees and Dutch controls in terms of six control variables.

| Variable | Statistical test | Descriptive statistic | | | | | |
| --- | --- | --- | --- | --- | --- | --- | --- |
|  |  | Adoptees | | | Dutch controls | | |
|  |  | N | % | M (SD) | N | % | M (SD) |
| Age | *t*_56_ = -0.24, *p* = 0.13 | 29 |  | 32 (5) | 29 |  | 32 (7) |
| Education | χ^2^_(3)_ = 2.21, *p* = 0.53 |  |  |  |  |  |  |
| VBO |  | 2 | 7 |  | 2 | 7 |  |
| MAVO |  | 8 | 28 |  | 7 | 24 |  |
| HAVO |  | 9 | 31 |  | 5 | 17 |  |
| VWO |  | 10 | 35 |  | 15 | 52 |  |
| Visit | χ^2^_(1)_ = 0.07, *p* = 0.79 |  |  |  |  |  |  |
| Yes |  | 16 | 55 |  | 15 | 52 |  |
| No |  | 13 | 45 |  | 14 | 48 |  |
| Visit ratio | *t*_56_ = 0.46, *p* = 0.65 | 29 |  | 0.01 (0.02) | 29 |  | 0.01 (0.02) |
| Language | *t*_56_ = 0.88, *p* = 0.38 | 29 |  | 2.8 (1.1) | 29 |  | 2.6 (1.0) |
| Sex | χ^2^_(1)_ = 1.87, *p* = 0.17 |  |  |  |  |  |  |
| Female |  | 21 | 72 |  | 16 | 55 |  |
| Male |  | 8 | 28 |  | 13 | 45 |  |

**Table S2**. Statistics for comparison between early-adopted and later-adopted subgroups in terms of six control variables.

| Variable | Statistical test | Descriptive statistic | | | | | |
| --- | --- | --- | --- | --- | --- | --- | --- |
|  |  | Early-adopted subgroup | | | Later-adopted subgroup | | |
|  |  | N | % | M (SD) | N | % | M (SD) |
| Age | *t*_27_ = -4.02, *p* < 0.001 | 14 |  | 28.4 (4.3) | 15 |  | 34.7 (4.2) |
| Education | χ^2^_(3)_ = 3.37, *p* = 0.34 |  |  |  |  |  |  |
| VBO |  | 0 | 0 |  | 2 | 13 |  |
| MAVO |  | 4 | 29 |  | 4 | 27 |  |
| HAVO |  | 6 | 43 |  | 3 | 20 |  |
| VWO |  | 4 | 29 |  | 6 | 40 |  |
| Visit | χ^2^_(1)_ = 0.91, *p* = 0.34 |  |  |  |  |  |  |
| Yes |  | 9 | 64 |  | 7 | 47 |  |
| No |  | 5 | 36 |  | 8 | 53 |  |
| Visit ratio | *t*_27_ = -0.05, *p* = 0.96 | 14 |  | 0.01 (0.02) | 15 |  | 0.01 (0.02) |
| Language | *t*_27_ = 0.14, *p* = 0.89 | 14 |  | 2.9 (1.0) | 15 |  | 2.8 (1.1) |
| Sex | χ^2^_(1)_ = 5.66, *p* < 0.05 |  |  |  |  |  |  |
| Female |  | 13 | 93 |  | 8 | 53 |  |
| Male |  | 1 | 7 |  | 7 | 47 |  |

**Table S3**. Correlation matrix between perceptual test results and production assessment results for (a) Korean adoptees and (b) Dutch controls.

(a) Korean adoptees

|  | **PERC.pre** | **PERC.mid** | **PERC.final** | **PERC.mid-pre** | **PERC.final-pre** | **PERC.final-mid** |
| --- | --- | --- | --- | --- | --- | --- |
| **IDEN.time1** | .407^*^ | .670^**^ | .642^**^ | .531^**^ | .501^**^ | -.039 |
| **IDEN.time2** | .369^*^ | .643^**^ | .681^**^ | .524^**^ | .574^**^ | .111 |
| **RATI.time1** | .460^*^ | .747^**^ | .683^**^ | .588^**^ | .516^**^ | -.116 |
| **RATI.time2** | .378^*^ | .665^**^ | .749^**^ | .545^**^ | .650^**^ | .217 |
| **IDEN.time2-1** | .019 | .086 | .182 | .091 | .206 | .221 |
| **RATI.time2-1** | .002 | .083 | .292 | .098 | .350 | .478^**^ |

(b) Dutch control participants

|  | **PERC.pre** | **PERC.mid** | **PERC.final** | **PERC.mid-pre** | **PERC.final-pre** | **PERC.final-mid** |
| --- | --- | --- | --- | --- | --- | --- |
| **IDEN.time1** | .143 | .114 | .144 | .031 | .067 | .039 |
| **IDEN.time2** | .195 | .147 | .393^*^ | .027 | .379^*^ | .349 |
| **RATI.time1** | .282 | .302 | .409^*^ | .196 | .315 | .141 |
| **RATI.time2** | .379^*^ | .369^*^ | .580^**^ | .199 | .468^*^ | .288 |
| **IDEN.time2-1** | .022 | .011 | .184 | -.007 | .248 | .251 |
| **RATI.time2-1** | .116 | .085 | .202 | .012 | .179 | .166 |

*Note:* **IDEN.time1**: identification accuracy for Time 1 productions, **IDEN.time2**: identification accuracy for Time 2 productions, **RATI.time1**: ratings for Time 1 productions, **RATI.time2**: ratings for Time 2 productions, **IDEN.time2-1**: improvement in identification accuracy from Time 1 to Time 2 productions, **RATI.time2-1:** improvement in ratings from Time 1 to Time 2 productions, **PERC.pre**: perceptual scores in pre-test, **PERC.mid**: perceptual scores in midway test, **PERC.final**: perceptual scores in final test, **PERC.mid-pre**: improvement from pre- to midway test, **PERC.final-pre**: improvement from pre- to final test, **PERC.final-mid**: improvement from midway to final test, *: *p* < 0.05, **: *p* < 0.01.

**Text S1: Materials and procedure for the childhood-vocabulary recognition test.**

For each trial, participants were presented with three different recordings of one Korean word. They were then presented with three Dutch words on a computer screen and asked to indicate which word they thought to be the correct translation. The test was self-paced.

Items consisted of 10 Korean words that at least 50% of Korean children have shown to comprehend by the age of 12 months (Pae & Kwak, 2011; using the MacArthur-Bates Communicative Development Inventory, henceforth CDI). A female native speaker of Korean recorded the word in a clear citation style in a soundproof booth. The Dutch words contained 10 correct translations and 20 alternatives (table 1 for all materials). The alternatives were Dutch translations of English words that at least 50% of American English children have shown to comprehend by the age of 12 months (Fenson et al., 1994): the Dutch CDI norms were not available at the time of preparing the study, so the English CDI norms were used instead.

The percentage correct was above chance for both adoptees (M=.46, *t*_29_ = 4.0, *p* < 0.001) and Dutch controls (M=.48, *t*_29_ = 7.5, *p* < 0.001), which might be due to prosody or the onomatopoeic nature of some of the Korean items.

**Text S1, Table 1.** Materials (and *English translations*).

| Korean word | Dutch word | | |
| --- | --- | --- | --- |
|  | Correct answer | Alternative 1 | Alternative 2 |
| mamma (*food*) | eten | bal (*ball*) | neus (*nose*) |
| kkakkung (*peekaboo*) | kiekeboe | luier (*diaper*) | op (*all gone*) |
| jjakjjakkung (*clap your hands*) | in je handen klappen | slaap lekker (*good night*) | dansen (*dance*) |
| manse (*hurray*) | hoera | fles (*bottle*) | mmm lekker (*yum yum*) |
| mokyok (*bath*) | badje | oh oh (*uh oh*) | baby (*baby*) |
| swi (*pee*) | plas | sap (*juice*) | kusje (*kiss*) |
| eungka (*poo*) | poep | melk (*milk*) | schoen (*shoe*) |
| hajima (don't do that) | niet doen | buiten (*outside*) | beker (*cup*) |
| jueo (*give*) | geef | hoi (*hi*) | boek (*book*) |
| jiji (*dirty*) | vies | knuffel (*hug*) | koekje (*cookie*) |

*References*

Fenson, L., Dale, P.S., Reznick, J.S., Bates, E., Thal, D.J., & Pethick, S.J. (1994). Variability in early communicative development. *Monographs of the Society for Research in Child Development,* 59, 1-185.

Pae, S., & Kwak, K. (2011). *한국판 맥아더-베이츠 의사소통발달 평가 전문가 지침서* [Korean MacArthur-Bates Communicative Development Inventories User's Guide and Technical Manual]. Seoul, Korea: Mind Press.

**Text S2: Analysis of identification errors.**

Identification error was classified according to six types: misclassifying lenis as fortis, lenis as aspirated, fortis as lenis, fortis as aspirated, aspirated as lenis, and aspirated as fortis. Proportions of each error type were calculated according to Group (Adoptees, Dutch controls, Korean controls) and Time (1, 2) (table 1). As shown in the table, the proportions of incorrect responses varied among error types but the relative proportions of the error types were highly similar across groups: for instance, error in misclassifying lenis as aspirated and fortis as lenis were the two most common error types overall, presumably for reasons of acoustic similarity.

**Text S2, Table 1**. Relative proportions (and standard errors) of six error types on identification task according to Group and Time.

|  | Adoptees |  |  | Dutch controls |  |  | Korean controls |
| --- | --- | --- | --- | --- | --- | --- | --- |
| Error types | Time 1 | Time 2 |  | Time 1 | Time 2 |  | Time 1 |
| Lenis as fortis | .128 (.018) | .149 (.025) |  | .144 (.020) | .133 (.022) |  | .157 (.036) |
| Lenis as aspirated | .259 (.025) | .275 (.028) |  | .235 (.030) | .217 (.028) |  | .386 (.041) |
| Fortis as lenis | .208 (.023) | .229 (.024) |  | .214 (.029) | .244 (.031) |  | .260 (.033) |
| Fortis as aspirated | .135 (.023) | .131 (.020) |  | .117 (.020) | .138 (.022) |  | .088 (.011) |
| Aspirated as lenis | .122 (.016) | .122 (.016) |  | .131 (.019) | .150 (.019) |  | .049 (.009) |
| Aspirated as fortis | .148 (.023) | .101 (.117) |  | .158 (.022) | .118 (.022) |  | .060 (.016) |
| Total | 1.0 | 1.0 |  | 1.0 | 1.0 |  | 1.0 |

Repeated-measures ANOVAs (separate for each time point, with Group and Error types as independent variables) indeed revealed significant main effects of Error types for Time 1 (*F*_5,280_ = 8.3, *p* < 0.001) and Time 2 production samples (*F*_5,280_ = 10.3, *p* < 0.001), with no interactions between Error types and Group. Paired t-tests were also carried out to compare the error proportions between samples at Time 1 and Time 2, separately for each group and each error type (i.e., 12 *t*-tests = 2 groups X 6 error types). Results showed no significant difference between Time 1 and Time 2, except for one type of error in misclassifying aspirated as fortis for which proportions decreased from Time 1 to Time 2 for both adoptees’ (*t*_28_ = 2.49, *p* < 0.05) and Dutch controls’ productions (*t*_28_ = 2.26, *p* < 0.05) (see table 1).


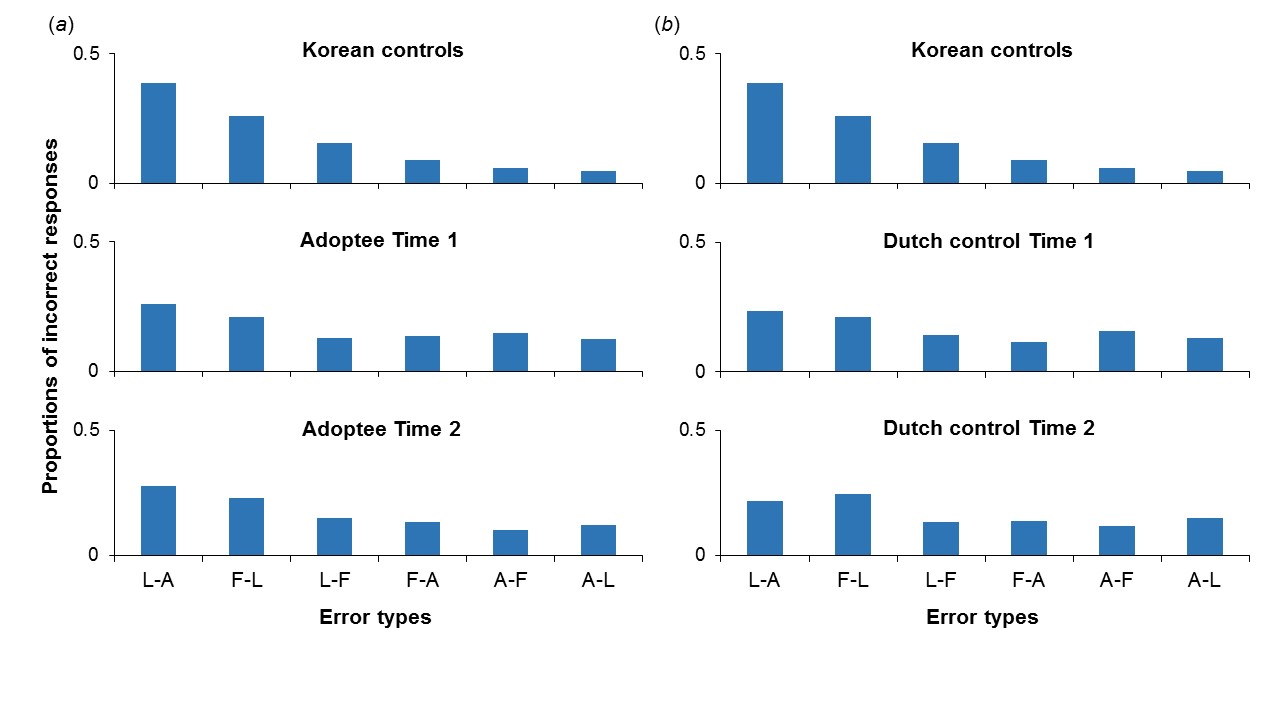


**Text S2, Figure 1.** Mean proportions of incorrect responses in identification task as a function of error types (a) for Korean adoptees’ productions at Time 1 and 2 in comparison to those for Korean controls’ productions, and (b) for Dutch controls’ productions at Time 1 and 2 in comparison to those for Korean controls’ productions (L-A, misclassifying lenis as aspirated; F-L, misclassifying fortis as lenis; L-F, misclassifying lenis as fortis; F-A, misclassifying fortis as aspirated; F-A, misclassifying fortis as aspirated; A-F, misclassifying aspirated as fortis; A-L, misclassifying aspirated as lenis).

The most common types of error for the Korean controls’ productions were again misclassification of lenis as aspirated and fortis as lenis. As shown in figure 1, the general pattern of errors was similar for all three groups’ productions. Spearman’s rho computed across the six error types showed, however, that from Time 1 to Time 2, the adoptees’ productions increased their correlations with the Korean controls’ productions (Time 1: *r_s_* = 0.77, *p* = 0.072; Time 2: *r_s_* = 0.94, *p* < 0.01), while those for the Dutch controls became less well correlated with the Koreans’ productions (Time 1: *r_s_* = 0.71, *p* = 0.111; Time 2: *r_s_* = 0.54, *p* = 0.266).

**Text S3: Control analyses.**

Correlations were calculated between the adoptees’ accuracy and rating scores in Time 1 and 2 productions on the one hand, and each control factor on the other hand (age, education, visits to Korea, visit ratio, languages), except for the binary variable sex where a *t*-test was used. Negative correlations were found between age at testing and the identification accuracy for both Time 1 and Time 2 productions (Time 1: *r* = -0.524, *p* = 0.004; Time 2: *r* = -0.367, *p* = 0.05), and also between age at testing and the ratings for both productions (Time 1: *r* = -0.496, *p* = 0.006; Time 2: *r* = -0.471, *p* = 0.01): the higher the adoptee’s current age, the lower their utterances were rated. Sex was also significantly related to the production scores, but only with the accuracy for Time 1 productions (*t*_27_ = 2.15, *p* = 0.041): the earliest productions of female speakers (M = 0.57) were significantly more accurately identified than those of male speakers (M = 0.48).

ANOVAs (by listener and speaker) on each dependent measure for the Dutch controls’ utterances, with the added variable Relationship, revealed no significant differences in any dependent measure between the Related and Unrelated control subgroups, except that, by listeners only, Time 2 productions of Unrelated controls received significantly higher accuracy (*F*_1,27_ = 8.0, *p* = 0.009) and ratings (*F*_1,27_ = 9.5, *p* = 0.005) than those of Related controls. These results indicate that the absence of a relationship did not lead to inferior control performance.

**Text S4: Correlations between perceptual test results and production assessment results from hierarchical regression analyses.**

Hierarchical regressions were carried out to examine correlations between perceptual test results and production assessment results while controlling for the influence of control factors. The control factors were entered into the first step of the regressions and the perceptual test results entered second. Note that a control factor of Visit was not entered because this factor was highly correlated with another control factor of Visit ratio (*r* = 0.60, *p* < 0.001): thus, five control factors (i.e., Age, Education, Visit ratio, Language, and Sex) were included. Table 1 below provides a summary of regression results.

**Text S4, Table 1.** A summary of hierarchical regression results for (a) Korean adoptees and (b) Dutch controls.

1. Korean adoptees

|  | ***R^2^*** | **PERC.pre** | | **PERC.mid** | | **PERC.final** | | **PERC.mid-pre** | | **PERC.final-pre** | | **PERC.final-mid** | |
| --- | --- | --- | --- | --- | --- | --- | --- | --- | --- | --- | --- | --- | --- |
|  |  | △*R^2^* | *Β* | △*R^2^* | *β* | △*R^2^* | *β* | △*R^2^* | *β* | △*R^2^* | *β* | △*R^2^* | *β* |
| **IDEN.time1** | .53* | .00 | -.09 | .14* | .45* | .11* | .44* | .18** | .47** | .15** | .44** | .01 | -.11 |
| **IDEN.time2** | .43 | .00 | .03 | .16* | .49* | .22** | .62** | .18** | .46** | .24** | .55** | .01 | .09 |
| **RATI.time1** | .45 | .02 | .17 | .23** | .59** | .16* | .52* | .21** | .49** | .12* | .40* | .04 | -.23 |
| **RATI.time2** | .42 | .00 | .05 | .15* | .47* | .64** | .62** | .16* | .43* | .23** | .54** | .01 | .13 |
| **IDEN.time2-1** | .33 | .01 | .16 | .01 | .15 | .08 | .36 | .01 | .08 | .05 | .26 | .07 | .29 |
| **RATI.time2-1** | .16 | .01 | -.13 | .00 | -.02 | .05 | .29 | .00 | .04 | .08 | .33 | .20* | .48* |

(b) Dutch control participants

|  | ***R^2^*** | **PERC.pre** | | **PERC.mid** | | **PERC.final** | | **PERC.mid-pre** | | **PERC.final-pre** | | **PERC.final-mid** | |
| --- | --- | --- | --- | --- | --- | --- | --- | --- | --- | --- | --- | --- | --- |
|  |  | △*R^2^* | *Β* | △*R^2^* | *β* | △*R^2^* | *β* | △*R^2^* | *β* | △*R^2^* | *β* | △*R^2^* | *β* |
| **IDEN.time1** | .46* | .05 | .25 | .00 | .05 | .00 | .08 | .02 | -.16 | .02 | -.19 | .00 | .00 |
| **IDEN.time2** | .42 | .02 | .17 | .00 | .05 | .09 | .39 | .01 | -.07 | .07 | .34 | .10 | .38 |
| **RATI.time1** | .33 | .07 | .31 | .05 | .25 | .11 | .43 | .01 | .12 | .03 | .22 | .00 | .07 |
| **RATI.time2** | .15 | .09 | .35 | .07 | .29 | .29* | .70** | .02 | .16 | .20* | .56* | .07 | .32 |
| **IDEN.time2-1** | .25 | .01 | -.10 | .00 | -.01 | .04 | .25 | .01 | .09 | .14* | .47* | .07 | .31 |
| **RATI.time2-1** | .25 | .00 | .05 | .00 | .06 | .06 | .31 | .00 | .05 | .09 | .37 | .05 | .27 |

*Note:* ***R^2^***: *R*-square for the first-step regression to show the degree of variance that is accounted for by control factors, **△*R***: the change in *R^2^* for the second-step regression to show the degree of unique variance that is accounted by perceptual test results, ***β***: standardized coefficients between perceptual test results and production assessment results while controlling for the control factors, **IDEN.time1**: identification accuracy for Time 1 productions, **IDEN.time2**: identification accuracy for Time 2 productions, **RATI.time1**: ratings for Time 1 productions, **RATI.time2**: ratings for Time 2 productions, **IDEN.time2-1**: improvement in identification accuracy from Time 1 to Time 2 productions, **RATI.time2-1:** improvement in ratings from Time 1 to Time 2 productions, **PERC.pre**: perceptual scores in pre-test, **PERC.mid**: perceptual scores in midway test, **PERC.final**: perceptual scores in final test, **PERC.mid-pre**: improvement from pre- to midway test, **PERC.final-pre**: improvement from pre- to final test, **PERC.final-mid**: improvement from midway to final test, *: *p* < 0.05, **: *p* < 0.01.

**Figure S1.**

**
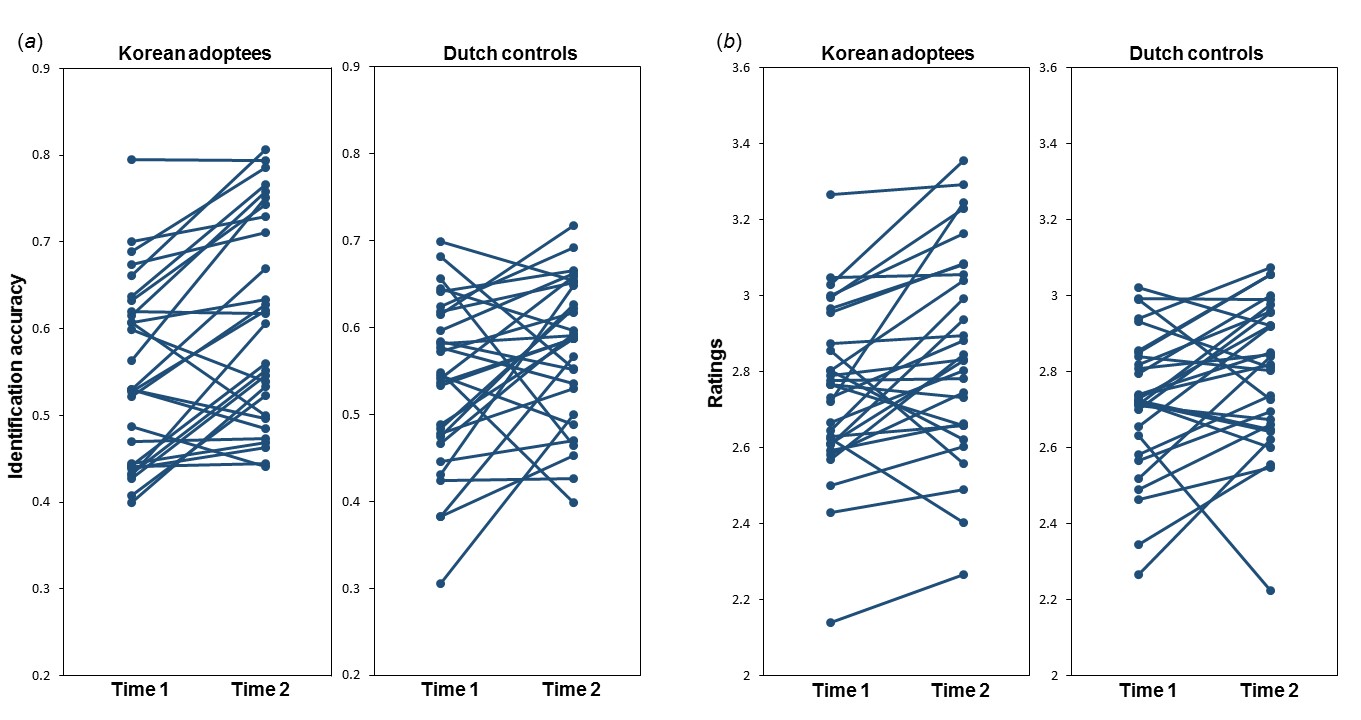
**

**Figure S1.** (a) Mean proportions of correct identification for Korean adoptees (left panel) and Dutch controls (right panel) as a function of production time, and (b) mean ratings for Korean adoptees (left panel) and Dutch controls (right panel) as a function of production time.
